# Supplementary figures and images for: Role of β-Caryophyllene in the Antinociceptive and Anti-Inflammatory Effects of Tagetes lucida Cav. Essential Oil
Source: Molecules. 2020 Feb 5;25(3):675. doi: 10.3390/molecules25030675 (PMC7037113; doi:10.3390/molecules25030675)

Instrument: JEOL GCmate

Inlet: GC

Ionization mode: EI+

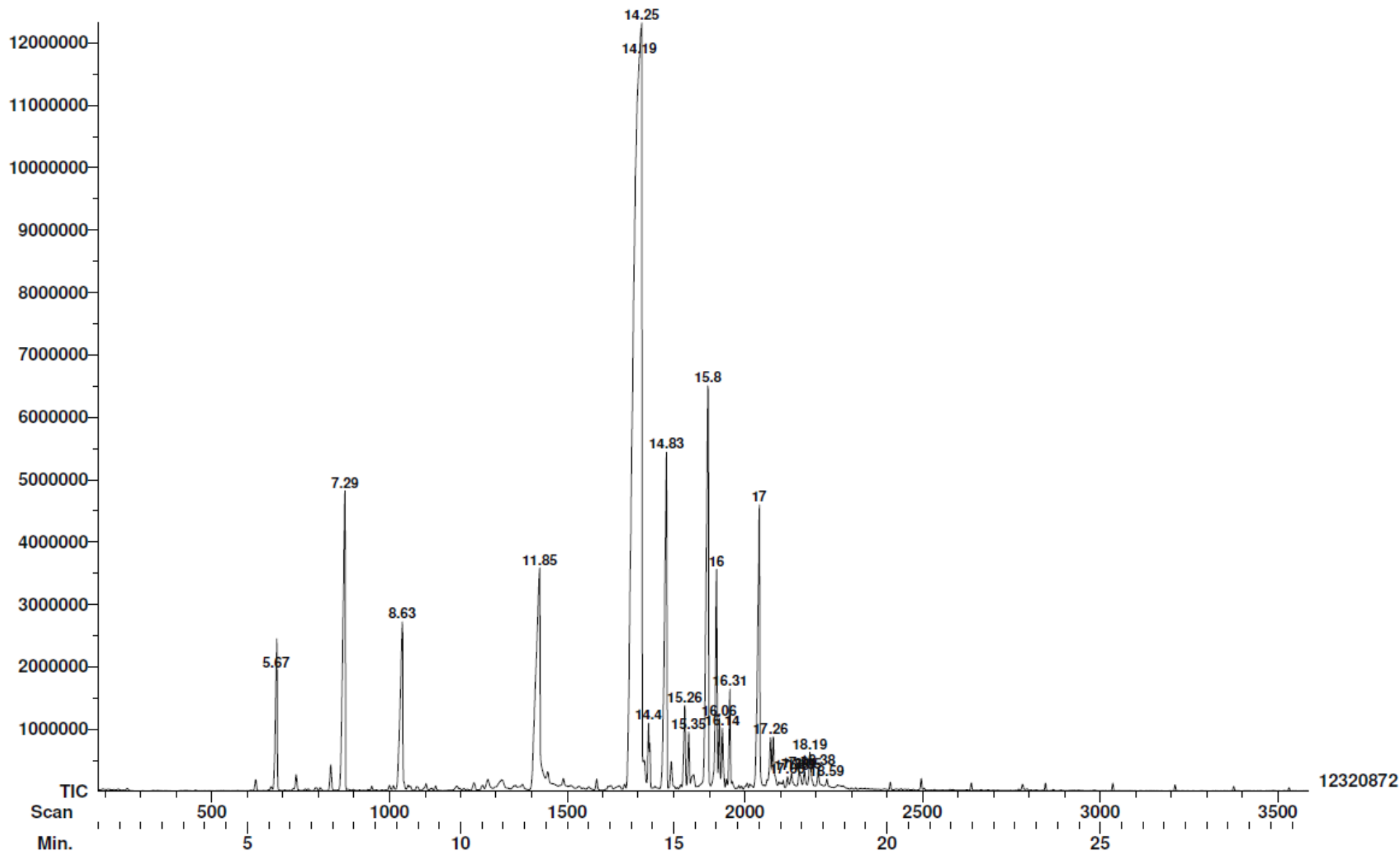

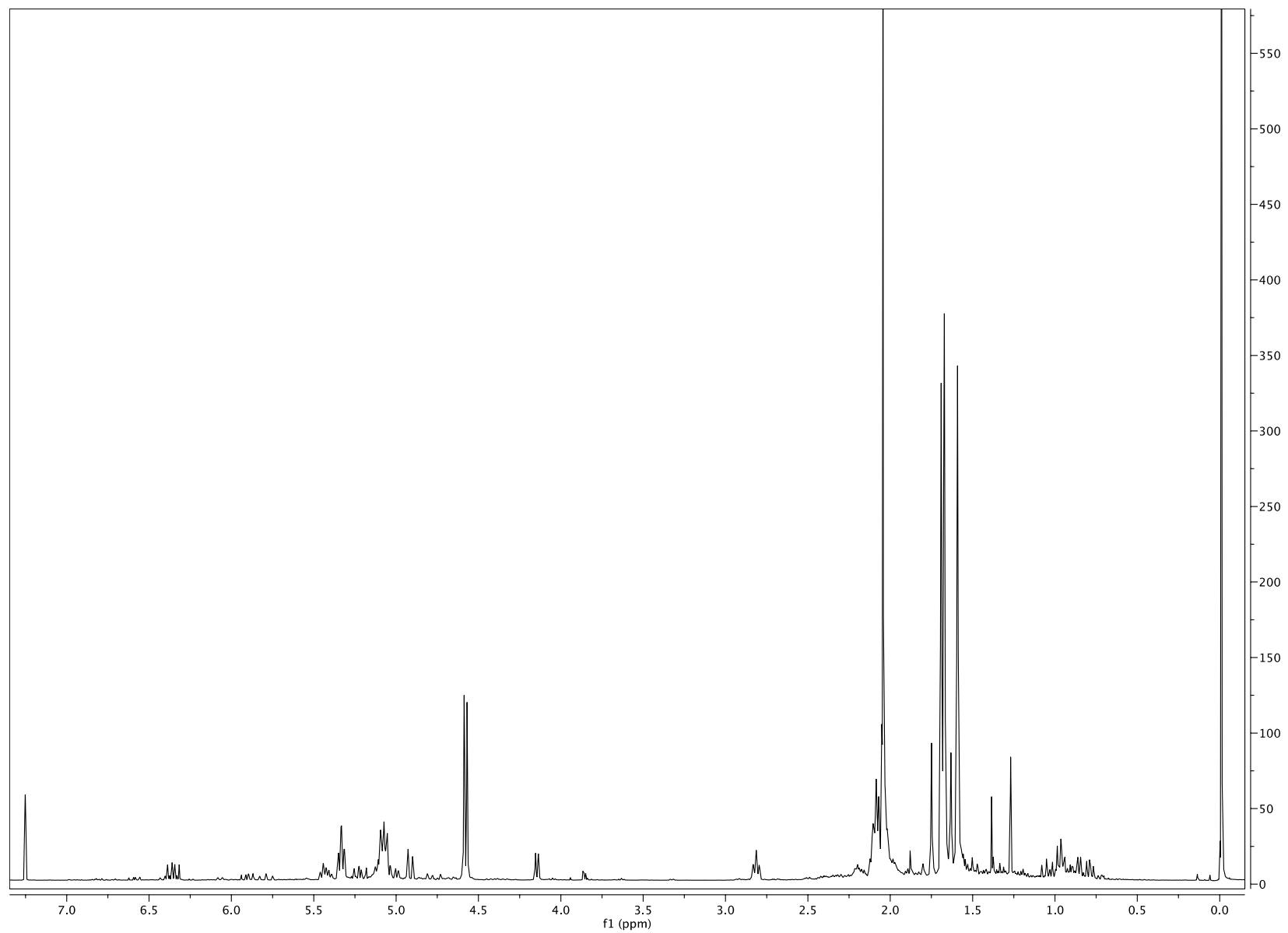

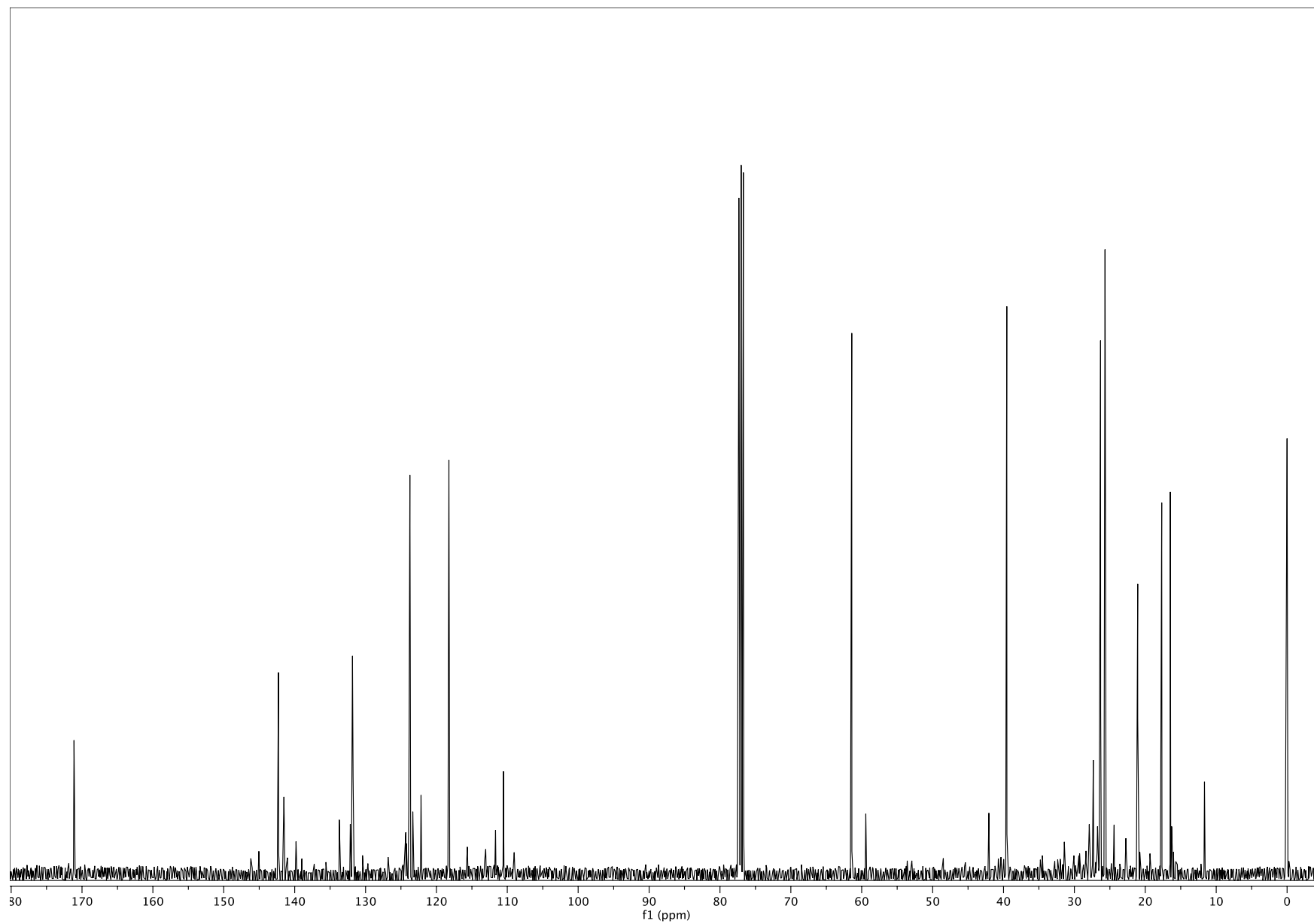

Supplement: Supplementary file 1 [file molecules-25-00675-s001.pdf]
